# Creativity’s Impact on Aging and Health, 1970–2010

**DOI:** 10.1093/geroni/igaa057.2209

**Published:** 2020-12-16

**Authors:** Wendy Miller

**Affiliations:** Create Therapy Institute, Kensington, Maryland, United States

## Abstract

In the 1970, practitioners in the arts, aging, health, social work and allied therapeutic professions became pioneers in a newly formed movement of creative aging. Robert Butler’s “Why Survive? Growing Old in America” called for rethinking aging and the services needed to support the growing aging population. Barriers to restrictions and the lower expectations around how older people could live in their communities began to change. Opportunities for engagement in the humanities and the arts began to be tailored to support older people’s life style and to accommodate the sharing of their life experiences in new ways through partnerships with schools and senior centers. The rise of these practices promoted the first multi-year study of arts engagement impact on the health on older adults and a wave of publications and new research studies followed. These were the foundation for this strength-based initiative across the spectrum of aging.

